# Benchmarking and improving the performance of variant-calling pipelines with RecallME

**DOI:** 10.1093/bioinformatics/btad722

**Published:** 2023-12-13

**Authors:** Gianluca Vozza, Emanuele Bonetti, Giulia Tini, Valentina Favalli, Gianmaria Frigè, Gabriele Bucci, Simona De Summa, Mario Zanfardino, Francesco Zapelloni, Luca Mazzarella

**Affiliations:** Department of Experimental Oncology, European Institute of Oncology IRCCS, Milan, Italy; Department of Oncology and Hematology-Oncology, Università degli Studi di Milano, Milan, Italy; Department of Experimental Oncology, European Institute of Oncology IRCCS, Milan, Italy; Department of Oncology and Hematology-Oncology, Università degli Studi di Milano, Milan, Italy; Department of Experimental Oncology, European Institute of Oncology IRCCS, Milan, Italy; 4bases SA, Manno, Ticino, Switzerland; Department of Experimental Oncology, European Institute of Oncology IRCCS, Milan, Italy; Center for Omics Sciences, IRCCS Ospedale San Raffaele, 20132 Milano, Italy; Molecular Diagnostics and Pharmacogenetics Unit, IRCCS Istituto Tumori, “Giovanni Paolo II”, Bari, Italy; IRCCS Synlab SDN, 80143, Naples, Italy; 4bases SA, Manno, Ticino, Switzerland; Department of Experimental Oncology, European Institute of Oncology IRCCS, Milan, Italy

## Abstract

**Motivation:**

The steady increment of Whole Genome/Exome sequencing and the development of novel Next Generation Sequencing-based gene panels requires continuous testing and validation of variant calling (VC) pipelines and the detection of sequencing-related issues to be maintained up-to-date and feasible for the clinical settings. State of the art tools are reliable when used to compute standard performance metrics. However, the need for an automated software to discriminate between bioinformatic and sequencing issues and to optimize VC parameters remains unmet.

**Results:**

The aim of the current work is to present RecallME, a bioinformatic suite that tracks down difficult-to-detect variants as insertions and deletions in highly repetitive regions, thus providing the maximum reachable recall for both single nucleotide variants and small insertion and deletions and to precisely guide the user in the pipeline optimization process.

**Availability and implementation:**

Source code is freely available under MIT license at https://github.com/mazzalab-ieo/recallme. RecallME web application is available at https://translational-oncology-lab.shinyapps.io/recallme/. To use RecallME, users must obtain a license for ANNOVAR by themselves.

## 1 Introduction

The analytical performance of novel Next Generation Sequencing (NGS)-based and Third Generation Sequencing (TGS)-based variant calling (VC) pipelines requires comparing the experimental dataset with a “ground truth” dataset of expected variants (Salit and Zook 2019).

A key step in benchmarking is variant harmonization, as discrepancies in variant representation between ground truth and experimental dataset may lead to incorrectly interpreting a variant as missing in the experimental dataset.

Based on initial accuracy quantification, VC pipelines can be further optimized by modifying calling parameters and thresholds for quality metrics. This may be necessary in situations with a different tradeoff between recall and specificity: although large research-oriented studies may prefer removing false positives at the expense of true positives, as this may be compensated by the power afforded by large sample sizes, priorities are inverted in the diagnostic setting, where in general maximal recall is preferred, since the analysis is conducted on individuals, it bears clinical and legal relevance and a false positive (FP) rate in key variants may be tolerated since these can be orthogonally validated, for instance by Sanger sequencing. In addition, especially for false negative (FN) calls, it is important to understand the source of miscalling, which can be due to two broad families of faults: those occurring during the “wet” part of the workflow (library preparation and sequencing), which can only be corrected by redesigning the analytical assay, and those associated with the bioinformatic pipeline, which can in theory be corrected by modifying the parameters of the “dry” workflow.

Currently, the standard software for pipeline benchmarking is hap.py ( Krusche *et al.* 2019) although its “simpler” version som.py is often used across several indications (both for germline and somatic VC) as it simply compares the presence of specific sequences at given positions between the ground truth and query callsets without attempting to match haplotype, which is in general problematic in cancer somatic sequencing. Som.py provides a workflow for variant harmonization and generates accuracy metrics, but suffers from specific limitations: (i) it does not provide information on quality parameters of variants identified as FN and FP; (ii) it has not been widely implemented on other VC pipelines, like Ion Torrent-based (ION) sequencing, common in clinical settings (Azzollini *et al.* 2023, Ricci *et al.* 2023, Schnidrig *et al.* 2023), for which the dominant VC algorithm is the Torrent Variant Caller (TVC). ION technology shows heterogeneity in variant annotation and some limitations in detecting insertion and deletion variants (INDELs) that may lead to incorrect estimates of the actual analytical performance (Loman *et al.* 2012, Laehnemann *et al.* 2016, Marine *et al.* 2020). Third-generation sequencing technologies like Oxford Nanopore Technologies are increasingly being used also in the diagnostic setting and thus require appropriate benchmarking tools, as ONT is able to resolve highly repetitive regions (Jain *et al.* 2022) but the detection of small variants remains challenging (Rang *et al.* 2018).

Here, we describe RecallME, a tool designed to standardize variant annotation across multiple callers, rapidly quantify performance metrics for NGS/TGS-based VC pipelines, discriminate between sequencing and bioinformatic errors and guide the user in the pipeline optimization process.

## 2 Materials and methods

### 2.1 Overview

RecallME was developed to identify and resolve the following drawbacks in accuracy assessment: (i) variant notation heterogeneity among callers; (ii) deconvolution of multi-allelic sites; (iii) identification of the cause for FN calls, through time-consuming re-check of supporting reads in the BAM file; and (iv) parameter optimization on recall and precision.

**Figure 1.**
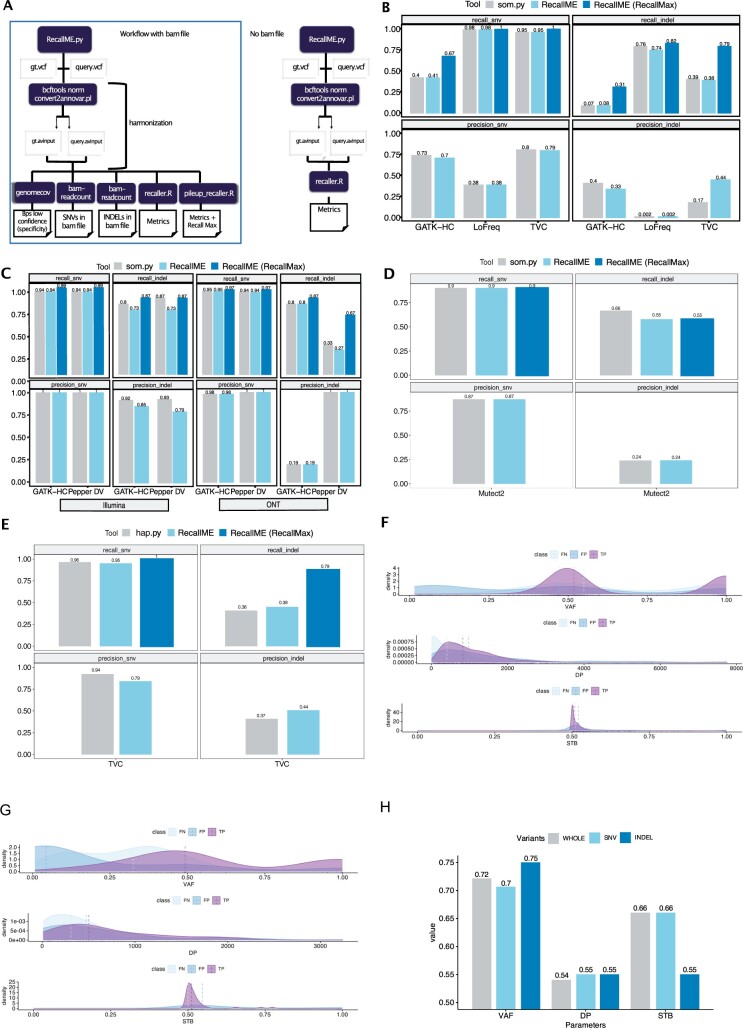
RecallME allows the maximization of the recall in reference samples. (A) Flowchart that shows how RecallME suite works with and without the bam file as input. First, bcftools norm splits the multi-allelic variants, then the query and the ground truth VCF files are converted in annovar inputs to harmonize variant notations. Bedtools genomecov function computes the number of bps that are not considered as high confidence by subtracting bps outside the bed file (to compute the number of true negatives and, consequently, the specificity). Bam-readcount look for SNVs and INDELs within the bam file to check if the recall can be maximized. The recaller.R and the pileup_recaller.R scripts compute the standard metrics as recall, precision, specificity, F1-score, FDR, and the Recall Max. (B) Barplots showing recall metrics in TVC and LoFreq pipelines (ION technology) and GATK-HC (Illumina-based) computed by som.py and RecallME (before and after BAM file re-check step) in SNV and INDEL calls in NA12878 sample. (C) Barplots showing recall metrics in GATK-HC and Pepper DV pipelines (Illumina and ONT-based) computed by som.py and RecallME (before and after BAM file re-check step) in SNV and INDEL calls in HD793 sample (Illumina and ONT). (D) Barplots showing accuracy metrics in Mutect2 pipeline computed by som.py and RecallME (before and after BAM file re-check step, i.e. RecallMax) in SNV and INDEL calls in SEQC2 somatic dataset. (E) Barplots showing recall metrics in TVC pipeline (ION technology) computed by hap.py and RecallME (before and after BAM file re-check step) in SNV and INDEL calls in NA12878 dataset. (F) TVC parameters distributions across TPs, FPs and b-FNs in SNV variants. Statistically significant differences have been found in VAF, DP, and STB (Mann–Whitney two-sided test). (G) TVC parameters distributions across TPs, FPs, and b-FNs in INDEL variants. Statistically significant differences have been found in VAF, DP, and STB (Mann–Whitney two-sided test). (H) Accuracies in TVC performances (NA12878) by tuning optimal cutpoints for VAF, DP, and STB for SNVs, INDELs and whole calls (WHOLE).

Figure 1A shows a standard RecallME workflow:

- variant harmonization: complex variants are decomposed with bcftools norm (Danecek *et al.* 2021) and harmonized (left-alignment and parsimonious reporting) between the query VCF and the ground truth VCF using the convert2annovar.pl function of ANNOVAR (Wang et al. 2010). This allows to split multiallelic sites into individual variants, maintaining the associated quality information and VC parameters and retaining variants with nonzero frequencies; the associated annotation is a desirable but nonessential outcome of this step
- accuracy quantification: standard accuracy metrics as recall, precision, false discovery rate, F1-score and specificity through the bedtools (Quinlan and Hall 2010) *genomecov* function are computed;
- BAM re-check: FNs are automatically re-evaluated in the BAM file using functions in the bam-readcount package (Khanna *et al.* 2022), which outputs a list of variants with variant caller-independent associated parameters as depth (DP), variant allele frequency (VAF) and percentage of strand-bias (STB). If a putative FN is found in the re-check step, we recalculate the recall metric as the maximum theoretical recall attainable (RecallMax, Supplementary Fig. S1) based on the available sequencing results (i.e. by assuming that a variant with supporting reads in the BAM file can be in principle called upon relaxing VC parameters);
- parameter visualization and accuracy simulation: thresholds for VC quality parameters can be fine-tuned through a R Shiny application (semantic.dashboard v.0.2.1, available at https://translational-oncology-lab.shinyapps.io/recallme/), for instance to identify VC thresholds that maximize precision or recall, depending on the purpose of the NGS panel. The interactive dashboard reports the dynamically changing lists of True Positives (TPs), FPs and FNs with the related standard metrics and the variants that have not been found within the BAM file. The lists and the performance metrics can be optimized directly within the dashboard by setting new VC parameters thresholds.

Datasets were analyzed with RecallME, som.py (v.0.3.15) and hap.py (v.0.3.9). Analysis with som.py was performed on datasets after variant harmonization through bcftools *norm -m-any* function using as input the query and the ground truth VCF files and the bed file of the panel to limit regions where standard metrics have to be computed and, finally, the FASTA reference files: hg19 for the internal and external datasets. The SEQC2 consortium dataset has been aligned to hg38.

### 2.2 Definition of benchmarking accuracy metrics

VC benchmarking studies have a potential for ambiguity in the language, since we have in fact three datasets to compare (the ground truth, the VC pipeline output, the benchmarking output) and terms describing accuracy metrics may refer to any of the possible comparisons. This potential ambiguity mostly arises in defining FNs and is related to the cause of miscalling. A FN is a variant that is expected but not observed, but as discussed previously, its absence from the experimental dataset may be: (i) sequencing-related: the variant is not sequenced at all, for instance due to complete lack of coverage or allelic dropout; (ii) bioinformatic: the variant is sequenced, but does not pass the filters imposed by the VC pipeline. It is potentially observed if VC filtering is completely removed; (iii) symbolic: the variant is sequenced and called, but it is represented in a different way than in the ground truth, generating a mismatch.

After resolving type 3 (symbolic) FNs through variant harmonization, the distinction between type 1 and 2 FNs is key to identify points of intervention. To measure type 2 (bioinformatic) error, we introduce a metric, RecallMax, which measures the maximum theoretical sensitivity achievable through changes in VC bioinformatic parameters. RecallMax is defined as

$$\mathrm{RecallMax}\;=\frac{\mathrm{TPs}+b-\mathrm{FNs}}{\mathrm{TPs}+\mathrm{FNs}}$$

where b-FNs (bioinformatic-FNs) indicates the FNs for which the BAM re-check step has found supporting reads.

We then define the set of sequencing-positive variants:

SeqPos=TPs+b−FN+FPs

which we use as a reference dataset to quantify the impact of individual sequencing quality metrics on variant “callability”, i.e. the accuracy in calling an actually sequenced variant. In SeqPos, we pose TPs + b - FN = 1 (i.e. positively callable variants) and FPs = 0 (i.e. variants that should be called as negative). SeqPos is used to build Receiver Operating Curves (ROC) with sequencing quality metrics (e.g. VAF, DP, STB) as explanatory variables. The Area Under the ROC (AUROC) is calculated to measure the predictive power of each sequencing metric; optimal cutoffs were calculated using the VAF, DP and STB metrics in the cutpointr R package.

Statistical analyses were performed the two-sided Mann–Whitney test (with *α* = 0.05) for continuous variables.

### 2.3 Generation of sequencing datasets

We tested the performance of RecallME on three sets of variants obtained by the standard Genome In A Bottle (GIAB) NA12878 (Zook *et al.* 2019) sample split in two dataset: an internal and an external validation datasets. The internal dataset was composed of a query callset called with GATK Haplotype Caller (GATK-HC) and a ground truth retrieved from the GIAB official ftp. The ground truth set was a publicly available version sequenced with the TruSeq panel from Illumina (https://ftp.ncbi.nlm.nih.gov/giab/ftp/data/NA12878/Nebraska_NA12878_HG001_TruSeq_Exome/, BAM file: NIST-hg001-7001-ready.bam; VCF file: NIST-hg001-7001-gatk-haplotype.vcf; BED file: TruSeq_exome_targeted_regions.hg19.bed) which covers 62 286 318 bps for benchmarking the GATK-HC pipeline and a re-sequenced NA12878 (purchased from the Coriell Institute for Medical Research) with an *in-house* designed gene panel based on ION technology (AmpliSeq-based panel) for benchmarking the TVC and LoFreq pipelines. Our custom ION panel is designed to cover cancer driver genes for a total of 2 214 732 bps of genomic space, with amplicon size 125–175 bps; a manuscript describing the panel specifications and performance is currently under preparation; details are available upon reasonable request. The overlap between the ION and Illumina panels and the high-confidence region is 1 391 907 bps.

Library preparation was carried out using Ion Ampliseq custom panel (Thermo Fisher Scientific) and 10 ng of DNA were used to prepare libraries using two different primer pools. After 12-cycle PCR amplification products were barcode ligated and purified (Agencourt AMPure XP beads). Libraries were equimolar pooled to 50 pM, amplified and enriched using the Ion Chef system with the Ion 550 Kit-Chef (Thermo Fisher Scientific) and sequenced on 550 chip in a 200 bp run using Ion S5 GeneStudio XL system (Thermo Fisher Scientific). The mean coverage was 995 (SD = 674) for ION (test set), 169 (SD = 138) for Illumina (ground truth).

The external validation was performed on the HD793 sample (Horizon Discovery Ltd., https://horizondiscovery.com/en/reference-standards/products/brca-germline-i-gdna), which was sequenced on Illumina and ONT workflows. For both, the DNA library was prepared using the HEVA pro kit (4bases SA). 100 ng of genomic DNA were fragmented using a digestion enzyme and the A-tailed using library preparation kit. Hybridization and capture of target regions were done according to an internal protocol in use at 4bases. Sequencing was done on Illumina ISEC100. The procedure for the ONT sequencing was similar but after the probe captures and the barcoding of sequences were end-repaired and A-tailed and the Nanopore adapters were ligated (nano adapter, 4bases SA). The sample was then purified and loaded on a MinION MK1B sequencer.

For benchmarking on somatic VC, we retrieved data from the SEQC2 consortium (Fang *et al.* 2021). We used the intersection between WGS_IL_1.bowtie.muTect2.vcf dataset (https://ftp-trace.ncbi.nlm.nih.gov/ReferenceSamples/seqc/Somatic_Mutation_WG/analysis/SNVs/vcfs/WGS/WGS_IL_1.bowtie.muTect2.vcf.gz) and the high confidence regions bed file (https://ftp-trace.ncbi.nlm.nih.gov/ReferenceSamples/seqc/Somatic_Mutation_WG/release/v1.2.1/High-Confidence_Regions_v1.2.bed). The computation of standard metrics has been performed with RecallME and som.py. For the comparison we used a ground truth generated by concatenation of high-confidence_sSNV_in_HC_regions_v1.2.vcf and high-confidence_sINDEL_in_HC_regions_v1.2.vcf for testing both SNV and INDEL calls (https://ftp-trace.ncbi.nlm.nih.gov/ReferenceSamples/seqc/Somatic_Mutation_WG/release/v1.2.1/high-confidence_sINDEL_in_HC_regions_v1.2.1.vcf.gz and https://ftp-trace.ncbi.nlm.nih.gov/ReferenceSamples/seqc/Somatic_Mutation_WG/release/v1.2.1/high-confidence_sSNV_in_HC_regions_v1.2.1.vcf.gz).

### 2.4 VC and benchmarking

For the internal test dataset, VC was performed using three different pipelines: GATK HaplotypeCaller v.2.8 (GATK-HC) (McKenna *et al.* 2010, DePristo *et al.* 2011, Van der Auwera *et al.* 2013), Lofreq v.2.1.3.1 (Wilm *et al.* 2012), and TVC, for the latters we kept default parameters.

For the HD739, VC was performed using GATK-HC and Pepper DeepVariant (Pepper DV) (Shafin et al. 2021).

Som.py and RecallME were run on the resulting VCFs and recall/precision metrics were extracted and compared separately for SNV and indels.

The analyses were performed on a High-Performance Computing (HPC) cluster (2 frontend machines with 24 cores and 128 GB ram and 12 computing nodes with 28 cores and 128 GB ram).

We then performed an external validation of RecallME in order to assure the reproducibility of our workflow on a different machine (Linux operating system with 8 cores and 32 GB ram).

## 3 Results

### 3.1 Comparison of RecallME versus som.py and computation of the RecallMax

Initial software implementation was run on the ION dataset (see Section 2). Figure 1B shows accuracy performances obtained by RecallME and som.py and the computation of the RecallMax metric.

Both som.py and RecallME yielded similar accuracy metrics, although RecallME estimated a markedly higher precision for TVC indels, which we attribute to the two-step variant notation normalization feature implemented in RecallME, which allows the inclusion of the TVC output format among those immediately compatible with RecallME analysis. RecallMax was greater for INDELs (up to 40.5% for TVC) than SNVs (up to 26.5% for GATK-HC).

Further validation of the RecallME workflow was carried out on a variant dataset generated in an independent laboratory on a separate sample with Illumina and ONT-based sequencing.

RecallME was slightly more stringent than som.py in the Illumina dataset, although RecallMax provided virtually identical metrics. On the ONT dataset, performance was very similar, however RecallMax identified a very high rate of b-FNs for PepperDV (Fig. 1C).

Finally, in order to provide a comparison in a dataset especially relevant for somatic VC, we tested RecallME on the SEQC2 dataset. Performances computed by RecallME and som.py are virtually identical except for INDEL recall, in which som.py provided higher values (0.66) than RecallME (0.55) (Fig. 1D).

### 3.2 Comparison of RecallME versus hap.py

We tested RecallME and hap.py on the original internal validation dataset TVC-based VC. Recall and precision were quite similar, although INDEL precision was quantified as higher by RecallME (0.44 versus 0.37) (Fig. 1E), perhaps expectedly since exact haplotype matching (more impactful for INDEL calls than SNV) is not required for RecallME to score a match. In addition, RecallMax for SNV was maximal, indicating that were, in fact, all sequenced (recall = 1) and 60 b-FN INDELs can indeed be identified in the BAM. The parameter distribution can guide threshold reset to increase recall.

### 3.3 RecallME for VC parameter optimization

A key feature of RecallME is the possibility to extract sequencing parameters associated with false calls and simulate changes in accuracy upon relaxing thresholds in these parameters. Distributions of key parameters like VAF, DP, and STB for TPs, b-FNs, and FPs in the ION dataset are shown in Fig. 1F and G. RecallME tests for significant differences in parameter distributions between FPs and TPs and b-FNs and TPs with a two-tailed Mann–Whitney test. We obtained for SNVs: VAF, p_SNV_ < 0.0001 for the comparison TPs versus FPs; DP p_SNV_ = 0.01 for TPs versus FPs and p_SNV_ < 0.0001 for TPs versus b-FNs; STB p_SNV_ < 0.0001 for TPs versus FPs. While for INDELs: VAF, p_INDEL_ < 0.0001 for both comparison TPs versus b-FNs and TPs versus FPs; DP, p_INDEL_ < 0.001 for TPs versus b-FNs; STB, p_INDEL_ < 0.01 for TPs versus FPs. Visualization and testing for these parameters are integrated in a RShiny-based application. It should be noted that some variant callers do not support the QD metric in the parameters. In such cases, RecallME computes an adaptation of the quality by depth provided by TVC (QD = 4 $\frac{\mathrm{Quality}}{\mathrm{DP}}$).

The set of all sequencing-positive variants (SeqPos) that can be theoretically called at the bioinformatic level given the sequencing results can be used to estimate the impact of individual sequencing quality parameters on accuracy (see Section 2) on variant “callability”, i.e. the accuracy in calling an actually sequenced variant. An example of this analysis is shown in Fig. 1H: of three sequencing parameters analyzed (VAF, DP, STB), VAF has the largest impact overall. Importantly, AUC is higher for INDELs than for SNVs. Consequently, estimated optimal cutpoints are different for SNV (0.3) versus INDELs (0.07) (Supplementary Fig. S2), suggesting that using differential VAF filtering SNV versus INDELs may achieve superior diagnostic accuracy.

## 4 Conclusion

We here present a bioinformatic tool that facilitates the process of accuracy estimation for NGS panels and VC pipelines.

Our approach to variant harmonization is similar to that employed by hap.py/som.py (Krusche *et al.* 2019), with decomposition/reconstruction from complex variants to multiple simpler variants and left-alignment and parsimony. Some differences arise in the handling of complex variants, which result in minor deviations in the metrics computation. In addition, the usage of bcftools norm and ANNOVAR enables RecallME to present the information in a format that is compliant with the format used by bam-readcount, which in turn allows RecallME to identify b-FNs (sequenced but miscalled by the VC pipeline) and extract associated sequencing metrics, useful to optimize the VC pipeline based on the context-dependent need to prioritize sensitivity or specificity. We believe this feature to be particularly useful especially during the design and validation phases of NGS panels aimed at clinical diagnosis, and indeed this was the initial motivation for developement. RecallME is primarily thought for benchmarking cancer somatic sequencing data, but can in principle be applied to germline datasets, which are also more commonly employed as standard references, although haplotype comparison is not featured.

Although it was primarily designed to prevent some specific shortcomings of ION sequencing, it can be useful also for benchmarking the accuracy of other sequencing platforms, including Illumina and ONT, as it facilitates and automatizes tedious steps of fine-tuning the variant-calling workflow. Moreover, similarly to ION sequencing, the detection of INDELs in highly repetitive regions in ONT-based pipelines remain challenging (Sarkozy *et al.* 2017) and RecallME can easily detect noncalled INDELs to improve the maximum achievable recall.

## Supplementary Material

btad722_Supplementary_Data

## Data Availability

The data underlying this article are protected by IP and will be shared on reasonable request to the corresponding author. Cell line/DNA sample NA12878 was obtained from the NIGMS Human Genetic Cell Repository at the Coriell Institute for Medical Research. A subset of the original internal validation dataset is available under the run_example/folder within the Github repository and the related bam file is downloadable by running the *run_example.sh* script.
