# Supplementary figures and images for: Benchmarking and improving the performance of variant-calling pipelines with RecallME
Source: Bioinformatics. 2023 Dec 13;39(12):btad722. doi: 10.1093/bioinformatics/btad722 (PMC10748785; doi:10.1093/bioinformatics/btad722)

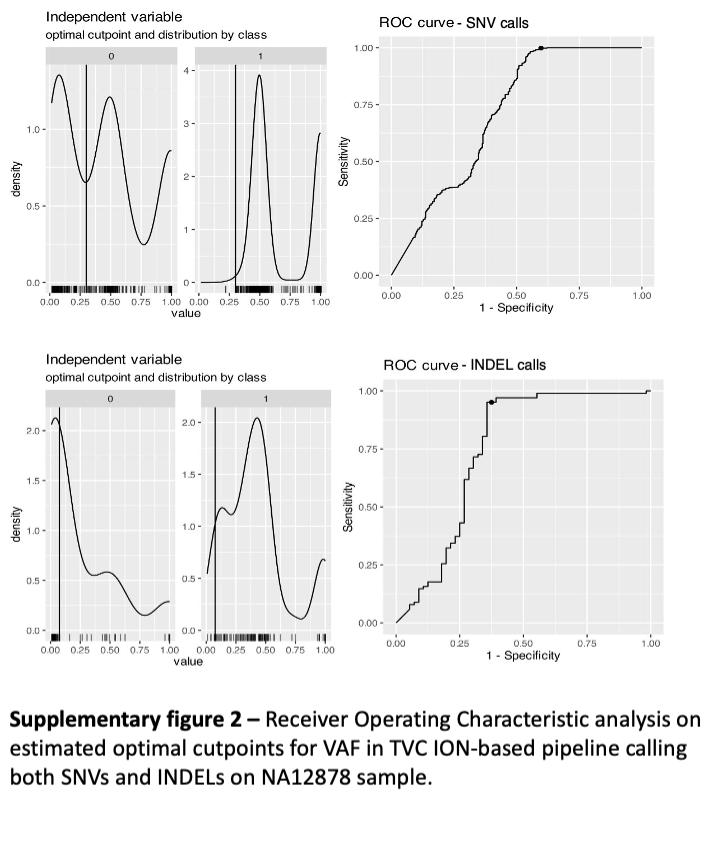

Supplement: btad722_Supplementary_Data [file btad722_supplementary_data.zip › Supplementary_figure2_RecallME.png]

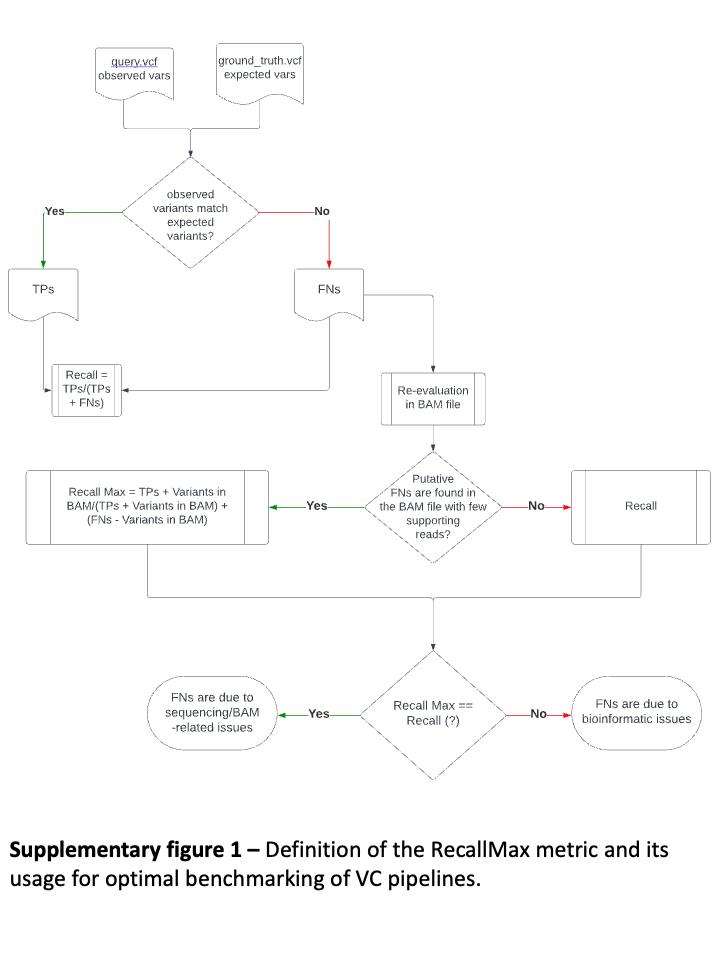

Supplement: btad722_Supplementary_Data [file btad722_supplementary_data.zip › Supplementary_figure1_RecallME.png]
